# New insights for mesospheric OH: multi-quantum vibrational relaxation as a driver for non-local thermodynamic equilibrium

**DOI:** 10.5194/angeo-36-13-2018

**Published:** 2018-01-09

**Authors:** Konstantinos S. Kalogerakis, Daniel Matsiev, Philip C. Cosby, James A. Dodd, Stefano Falcinelli, Jonas Hedin, Alexander A. Kutepov, Stefan Noll, Peter A. Panka, Constantin Romanescu, Jérôme E. Thiebaud

**Affiliations:** 1 Center for Geospace Studies, SRI International, Menlo Park, California, USA; 2 formerly at: Molecular Physics Laboratory, SRI International, Menlo Park, California, USA; 3 Air Force Research Laboratory (AFRL), Space Vehicles Directorate, Kirtland Air Force Base, New Mexico, USA; 4 Department of Civil and Environmental Engineering, University of Perugia, Perugia, Italy; 5 Department of Meteorology (MISU), Stockholm University, Stockholm, Sweden; 6 formerly at: Physical Sciences Division, SRI International, Menlo Park, California, USA; 7 The Catholic University of America, Washington DC, USA; 8 NASA Goddard Space Flight Center, Greenbelt, Maryland, USA; 9 Institute for Astro- and Particle Physics, University of Innsbruck, Innsbruck, Austria; 10 Institute of Physics, University of Augsburg, Augsburg, Germany; 11 German Remote Sensing Data Center (DFD), German Aerospace Center (DLR), Oberpfaffenhofen, Germany; 12 Aeris Technologies, Redwood City, California, USA

**Keywords:** Atmospheric composition and structure (air-glow and aurora; middle atmosphere composition and chemistry), history of geophysics (atmospheric sciences)

## Abstract

The question of whether mesospheric OH(*υ*) rotational population distributions are in equilibrium with the local kinetic temperature has been debated over several decades. Despite several indications for the existence of non-equilibrium effects, the general consensus has been that emissions originating from low rotational levels are thermalized. Sky spectra simultaneously observing several vibrational levels demonstrated reproducible trends in the extracted OH(*υ*) rotational temperatures as a function of vibrational excitation. Laboratory experiments provided information on rotational energy transfer and direct evidence for fast multi-quantum OH(high-*υ*) vibrational relaxation by O atoms. We examine the relationship of the new relaxation pathways with the behavior exhibited by OH(*υ*) rotational population distributions. Rapid OH(high-*υ*) + O multi-quantum vibrational relaxation connects high and low vibrational levels and enhances the hot tail of the OH(low-*υ*) rotational distributions. The effective rotational temperatures of mesospheric OH(*υ*) are found to deviate from local thermodynamic equilibrium for all observed vibrational levels.

## 1 Introduction

The emission of radiation from vibrationally excited OH is an important observable in the Earth’s upper atmosphere and has been the topic of numerous studies over the past several decades. This emission dominates the visible and infrared emissions from the atmosphere in this altitude region and has been used to investigate atmospheric density changes, temperature fluctuations, waves, tides, and species concentrations. Only a fraction of OH(*υ*) radiates; collisional energy transfer between OH(*υ*) and other atmospheric constituents significantly influences this emission and the mesospheric heat budget. Laboratory studies and theoretical calculations have investigated the relevant collisional energy transfer processes because they play a key role in determining the observed vibrational population distributions and their relative emission intensities. Modeling calculations of these emissions have also attracted considerable interest and are an essential part of the synergistic interplay between observations, laboratory experiments, and theoretical calculations.

In 1948, Aden Meinel reported complex near-infrared emissions in night sky spectra recorded using a grating spectrograph at the Lick Observatory, but these features remained unidentified at first (Meinel, 1948, 1950a). Meinel observed similar intense emissions at the Yerkes Observatory and, following the suggestion of Gerhard Herzberg (Herzberg, 1951), attributed them to OH rovibrational transitions within the electronic ground state (Meinel, 1950b, c, d). These emissions, known as the OH Meinel bands, represent some of the most prominent features in the visible and infrared regions of the nightglow. The overall emission exhibits an intensity peak near 87 km with a full width at half maximum of ~ 8 km (Sivjee, 1992; McDade, 1991; Meriwether, 1989; Baker and Stair, 1988; Evans et al., 1973). The peaks of the emission profiles of the individual vibrational levels exhibit modest altitude dependence with an upward shift of ~ 0.5 km for each increasing vibrational quantum number. This altitude dependence has been the topic of several investigations (e.g., von Savigny and Lednytskyy, 2013; von Savigny et al., 2012; Liu and Shepherd, 2006; McDade, 1991; López-Moreno et al., 1987).

The mesosphere represents a transition region between viscous flow and turbulent transport in the layers below and free molecular flow and diffusion above it. In the rarified environment of the mesosphere, the collision frequency is relatively low and it is not always possible for excited species to attain thermodynamic equilibrium, especially at the higher altitudes. For OH in particular, the question of whether the rotational temperature determined by observations is equivalent to the local kinetic temperature has been debated since the discovery of the Meinel band emission in the 1950s. This question is a matter of profound fundamental importance for our understanding of mesospheric chemistry and dynamics and for the interpretation of the variability of OH emissions. If thermodynamic equilibrium is attained, the observed emissions can be considered a reliable proxy that monitors temperature variability and mesospheric heat deposition. If not, then the determined OH rotational quasi-temperatures and their changes are controlled by the relevant altitude-dependent production, removal, and energy transfer processes.

The structure of the paper is as follows. First, we introduce the sources and sinks for the mesospheric vibrationally excited hydroxyl radical OH(*υ*). Then, we briefly review the history of the debate on the equivalence of the OH(*υ*) rotational temperatures and local kinetic temperature and the relevant atmospheric observations deriving rotational temperatures in the second half of the last century. The next two sections present significant relevant developments during the past decade based on ground-based observations of the OH Meinel band emission using astronomical telescopes and laboratory studies investigating the multi-quantum relaxation of OH(*υ*) by O atoms. We then present a comparison of results from observations at three different telescope sites, which provide unequivocal evidence that thermalization is incomplete for all observed OH vibrational levels. The available laboratory studies on OH rotational relaxation corroborate this conclusion. Moreover, we consider multi-quantum vibrational relaxation processes with emphasis on the recent developments for OH(*υ*) + O and examine the role of these processes in generating low vibrational levels with rotational excitation. Finally, we briefly discuss the implications of the new insights for our understanding of the mesosphere and future needs for relevant observations, laboratory measurements, and modeling calculations.

## 2 Mesospheric OH sources and sinks

### 2.1 Production by H +O_3_

The main source of mesospheric OH(*υ*) is the H + O_3_ reaction, as initially suggested by Bates and Nicolet (1950a, b) and Herzberg (1951). This reaction has a large exothermicity of 3.38 ev and is characterized by a strongly inverted internal state distribution of product OH. The majority of the available energy is channeled into OH internal degrees of freedom, with significant rotational excitation and more than ~ 90 % of the nascent product OH appearing in vibrational levels *υ* = 7–9 (Klenerman and Smith, 1987; Ohoyama et al., 1985; Charters et al., 1971). The most probable vibrational level is *υ* = 9, which is also the highest energetically allowed level. The main source of the O_3_ molecules is three-body recombination O + O_2_+ *M* (*M* = N_2_, O_2_). Photodissociation of water is the main source of hydrogen atoms that form a thin layer in the mesosphere, as originally pointed out by Bates and Nicolet (1950a, b).

### 2.2 Removal by radiative decay and rotational–vibrational relaxation

The OH Meinel band emission originates from radiative transitions between different rovibrational levels of the OH ground state. These transitions redistribute the vibrational and rotational level populations. The relevant radiative transition probabilities have evolved during the past decades and do not represent as significant a source of uncertainty as they did in the early years after the discovery of the OH emission. The most recent calculations (Brooke et al., 2016; van der Loo and Groenenboom, 2007, 2008) appear to be converging (Slanger, 2016). A thorough critical evaluation considering comparisons of all the recent sets of radiative transition probabilities is still needed to provide relevant guidance for future studies.

Aside from radiative transitions, reactions and collisional relaxation are responsible for the removal or redistribution of the nascent OH vibrational and rotational population distributions. Vibrational relaxation by molecular oxygen in the mesosphere is the dominant loss process for most altitudes and a significant fraction proceeds via multi-quantum pathways (Adler-Golden, 1997; Shalashilin et al., 1995; McDade, 1991). Laboratory experiments and theoretical calculations are needed to elucidate the details of multi-quantum vibrational relaxation pathways involving OH(*υ*).

In contrast to O_2_, vibrational relaxation by N_2_ is extremely inefficient and is dominated by single-quantum vibrational relaxation (Adler-Golden, 1997). Loss of vibrationally excited OH by atomic oxygen has been rather poorly understood and represents a major source of uncertainty for understanding and modeling mesospheric nightglow. Because of the importance of O atoms, we discuss in more detail recent relevant developments from laboratory experiments in Sect. 4.

Regarding the rotational equilibration of excited OH, the role of both major atmospheric constituents N_2_ and O_2_ is crucially important, with N_2_ dominating because of its larger abundance. In laboratory experimental studies, Holtzclaw et al. (1997) investigated the collisional relaxation of rotationally excited OH(*υ* = 1–3) by O_2_ at 100 K. They found that their observations could be reproduced by a model in which only transitions with Δ*N* = ± 1 were considered. Based on the state-to-state rotational energy transfer rate constants determined for rotational levels *N* = 8–25, a bottleneck in the population flow was established for *N* = 14. Kliner and Farrow (1999) performed experiments studying OH(*υ* = 0) in rotational levels *N* = 1–12 and determined that rotational relaxation by O_2_ and N_2_ is more efficient for lower rotational levels than for higher ones. They also found that an exponential gap model successfully reproduced their measurements and that translational relaxation of the nascent OH occurred much faster than rotational relaxation. No information is available on how collisions of OH with atomic oxygen may affect the hydroxyl rotational excitation, for example in the case that reactive or energy transfer processes have cross sections that vary as a function of the initial OH rotational level.

## 3 The long-standing debate on whether OH rotational temperatures are in LTE

Meinel (1950b) recognized that his observations of resolved OH spectral features lent themselves favorably to the determination of spectroscopic temperatures. He explored a variety of approaches, including analysis of transitions involving low rotational levels of the resolved *P* branch and the total relative intensities of the *P*, *Q*, and *R* branches of individual vibrational bands. He also found the rotational temperature of *υ* = 9 to be similar to that of *υ* = 4 and interpreted this finding as an indication that the rotational and ambient kinetic gas temperatures were equivalent (Meinel, 1950d). Nevertheless, an important conclusion from Meinel’s early work was that accurate results required quantitative knowledge of the OH excitation mechanism and the OH Meinel band absolute transition probabilities.

Other notable early studies (Wallace, 1961, 1962; McPherson and Vallance-Jones, 1960; Kvifte, 1959) supported the notion that consistent rotational temperatures in local thermodynamic equilibrium (LTE) could be obtained from the steady-state OH population. A key argument was that near the altitude of the emission layer each excited OH radical undergoes numerous collisions before radiating, and thus one can assume that the OH rotational temperature is in LTE. Chamberlain (1995) summarized the situation as follows: “… although this conclusion is not definitely established, it is reasonable to suppose that the rotational temperatures are indicative of the gas-kinetic temperatures.”

A vigorous debate ensued over several decades, with studies occasionally indicating discrepancies in the extracted rotational temperatures. Some researchers reported that the OH(*υ*) rotational temperatures appeared to increase with the vibrational level (Khomich et al., 2008, and references therein; Perminov et al., 2007, and references therein; Suzuki and Tohmatsu, 1976; Krasovskij and Šefov, 1965; Shefov, 1961). Shefov (1961) suggested that there are significant deviations from LTE for OH(high-*υ*) rotational levels with quantum number *N* > 5. However, the relatively poor signal, limitations in the available Einstein transition probabilities, active parallel debates on the effects of intensity, latitude, season of the year, and possibly the widespread consensus in favor of LTE limited the confidence in these results (Shefov, 1972a, b).

Other studies presented additional examples in which rotational features could be explained by non-equilibrium conditions (Gattinger and Vallance Jones, 1973; Harrison et al., 1971). Nichols et al. (1972) considered the effect of collisional relaxation and the possibility of emission from a non-thermalized rotational population distribution. Vallance Jones (1973) reviewed the available arguments from both sides, but could not reach a definitive conclusion. He suggested that dynamical effects might also influence mesospheric OH. Suzuki and Tohmatsu (1976) considered a collection of measurements from the literature and claimed that the rotational temperatures exhibit a dependence on the OH vibrational level from which the emission originates. This conclusion raised doubts because the comparisons of Suzuki and Tohmatsu involved selective data sets from different times and locations and also because different data collections did not appear to corroborate similar trends (Dick, 1977; Krassovsky et al., 1977). The main argument refuting the claim of Suzuki and Tohmatsu emphasized the fact that temperature differences can be reliably established only by simultaneous measurements. Otherwise, the temporal and spatial variations of the emissions and the uncertainties and possible systematic bias of different techniques hinder meaningful conclusions.

Reports indicating non-equilibrium conditions for OH continued appearing in the literature until the end of the last century. Pendleton et al. (1989) reported the observation of OH Meinel (7,4) band emission from rotational level *N* = 13. The column emission rates determined were estimated to be approximately 4 orders of magnitude larger than what would be expected under LTE. That work was later expanded with observations from vibrational levels *υ* = 3–7, providing additional evidence for incomplete thermalization of OH(*υ*) rotational excitation (Pendleton et al., 1993). Perminov and Semenov (1992) reported non-equilibrium intensities for transitions involving rotational levels *N* = 6–9 of the (7,3) band.

Finally, a series of studies by Dodd and coworkers using data from the Cryogenic Infrared Radiance Instrumentation for Shuttle (CIRRIS) aboard Space Shuttle mission STS-39 provided evidence for extremely high rotational excitation, up to ~ 2.3 eV in rotational energy. These studies reported results for several rotational levels of OH(*υ* = 0–9). Quite remarkably, spectra of pure rotational transitions for the four lowest vibrational levels, *υ* = 0–3, indicated population in rotational levels up to *N* = 33 (Dodd et al., 1993; Smith et al., 1992). Dodd et al. (1994) developed a model for the observed OH(*υ*, *N* ) column number densities and found that additional production of OH(low-*υ*) was required to match the observations. This work also concluded that, based on the Δ*N* = 0, ±1 selection rule for dipole-allowed transitions, the radiative cascade from nascent rovibrational levels cannot account for the observed emissions from the highest rotational levels of OH(*υ* = 0–3). The authors suggested two possible explanations for the enhanced rotational excitation: direct excitation by the H + O_3_ reaction or resonant vibrational-to-rotational energy transfer from nascent OH(*υ*) following collisions with O atoms. In a subsequent experimental study, Dodd and coworkers were able to detect rotationally excited OH(*υ* = 0, 1) from the reaction of O_3_ with fast H atoms. Nevertheless, the laboratory evidence led to the conclusion that the OH(*υ* = 0, 1) yield is very small compared to that of the high vibrational levels *υ* = 7–9 (Dodd et al., 1999).

Despite the aforementioned indications of non-LTE behavior in the observed OH(*υ*) rotational distributions, a common practice in the aeronomy community has been to assume that the rotational temperatures of isolated vibrational levels, obtained using a couple of selected spectral lines from low rotational quantum numbers, are in LTE and reflect the local kinetic temperature.

## 4 Recent developments on the vibrational relaxation of OH(high-*υ*) by O atoms

The removal of OH(high-*υ*) by O involves several pathways, including reaction to produce H + O_2_, single-quantum vibrational relaxation, and multi-quantum vibrational relaxation.

(1) $$\text{OH}(v=9)+O\to H+O_{2}\;\to \text{OH}(v=8)+O\;\to \text{OH}(v<8)+O$$

Laboratory measurements showed that the deactivation of OH(high-*υ*) by O(^3^*P* ) atoms is surprisingly fast, with a total removal rate constant of (4 ± 1) × 10^−10^ cm^3^ s^−1^ for OH(*υ* = 9) + O at room temperature (Kalogerakis et al., 2011). For the lower temperatures relevant to the mesosphere, we can estimate a value of (3 ± 1) × 10^−10^ cm^3^ s^−1^ if we assume that the temperature dependence of OH(*υ* = 9) + O is the same as that reported for experiments studying OH(*υ* = 7) + O (Thiebaud et al., 2010). These large values for the total removal rate constant approach the gas kinetic limit and defied an explanation until recently.

In 2015, Sharma et al. (2015) proposed that the interaction of OH(*υ*) with O atoms also involves a fast, spin-allowed, multi-quantum vibration-to-electronic (V–E) energy transfer pathway:

(2) OH(v≥5)+O(P3)→OH(0≤v′≤v-5)+O(D1).

Recent experiments (Kalogerakis et al., 2016) investigated this new pathway for OH(*υ* = 9) and provided laboratory evidence for rapid interconversion of the type

(3) OH(v=9)+O(P3)⇄OH(v=3)+O(D1).

In those experiments, an ultraviolet laser was used to photodissociate O_3_ in mixtures containing a small amount of H_2_ in Ar bath gas. Under the conditions employed, deactivation of O(^1^*D*) was relatively inefficient and OH(*υ* = 3) was produced in significant amounts from the photon-initiated reaction of O(^1^*D*) with H_2_. At the high laser energy used, the dissociation of O_3_ was practically complete and there-fore minimized the importance of the H + O_3_ reaction as a source of OH(*υ* = 9). A second tunable dye laser pulse was used to detect transient production of OH(*υ* = 9) arising from electronic-to-vibrational (E–V) energy transfer from O(^1^*D*) to OH(*υ* = 3).

The new multi-quantum V–E relaxation pathway was found to be the most efficient process for the deactivation of OH(*υ* = 9) by O atoms and provides an explanation for the surprisingly large increase in the rate constant of OH(*υ*) + O by more than 1 order of magnitude between *υ* = 0 (Burkholder et al., 2015, and references therein) and *υ* = 9 (Kalogerakis et al., 2011). Moreover, this relaxation pathway explains why previous theoretical calculations (Caridade et al., 2013; Varandas, 2004) yielded substantially smaller total removal rate constants for OH(high-*υ*) + O than the experimentally measured ones. Based on the calculations by Varandas (2004) for the reactive pathway (1) and for single-quantum relaxation, the experimentally measured value for the OH(*υ* = 9) + O total removal rate constant is more than 4 times larger than the theoretical rate constant for reaction and approximately 1 order of magnitude larger than the calculated value for single-quantum vibrational relaxation. A new generation of theoretical calculations involving excited potential energy surfaces will be necessary for experimental and theoretical results to converge. Laboratory measurements are also needed to quantify the relative importance of single-quantum and multi-quantum relaxation for atomic and molecular oxygen.

Finally, we note that Sharma et al. (2015) showed that the OH(*υ*) + O multi-quantum vibrational relaxation of Eq. (2) ultimately results in enhanced CO_2_ 4.3 μm emission. This enhancement involves energy transfer from O(^1^*D*) to N_2_, deactivation of the resulting N_2_(*υ* ≥ 2) by N_2_(*υ* = 0) to produce N_2_(*υ* = 1), and vibrational energy transfer from N_2_(*υ* = 1) to the *υ*_3_ mode of CO_2_, which promptly emits a 4.3 μm photon. Just as important, Panka et al. (2016, 2017a, b) recently implemented the Sharma mechanism in model calculations that resulted in very good agreement with observations of the nighttime CO_2_(*ν*_3_) 4.3 μm and the OH Meinel band emissions from the Sounding of the Atmosphere using Broadband Emission Radiometry (SABER) instrument aboard the NASA Thermosphere, Ionosphere, Mesosphere Energetics and Dynamics (TIMED) satellite.

## 5 Recent developments from observations by astronomical telescopes and evidence for non-LTE

During the last decade, renewed interest in exploiting the capabilities of astronomical telescopes has provided the most recent relevant information on OH(*υ*) rotational distributions (Noll et al., 2015, 2016; Oliva et al., 2015; Cosby and Slanger, 2007). The seminal studies of Cosby and Slanger (2007) and Noll et al. (2015) examined mesospheric OH(*υ*) in great detail using high-resolution sky spectra. Most important, these studies fulfill the crucial requirements of simultaneous, quantum-state-resolved measurements for all available vibrational levels.

Cosby and Slanger (2007) and Noll et al. (2015) examined the variability of mesospheric OH(*υ*) using data from astronomical telescopes. The former work used data from the High-Resolution Echelle Spectrograph (HIRES) on the Keck I telescope, the Echelle Spectrograph and Imager (ESI) on the Keck II telescope, and the UV-visual Echelle Spectrograph (UVES) of the Very Large Telescope (VLT) in Paranal, Chile, while the latter used data from the X-Shooter echelle spectrograph of the VLT. Both groups reported that the rotational temperatures determined from transitions involving the lowest rotational levels exhibit a clear vibrational level dependence, with the rotational temperature increasing by approximately 15 K as the OH vibrational quantum number increases from *υ* = 2 to *υ* = 8 (Noll et al., 2015; Cosby and Slanger, 2007). They also found that OH(*υ* = 8) has a significantly higher rotational temperature than OH(*υ* = 9).

The two aforementioned high-resolution data sets were obtained by different groups and instruments and at different locations and times. The trend in the overall behavior of the OH(*υ*) rotational temperatures as a function of the vibrational level persists even when different sets of transition probabilities are used in the analysis. This behavior can be considered an indication of non-LTE behavior because the mesopause and its associated temperature minimum occur several kilometers higher (~ 5–10 km) than the OH layer. Because the altitude of each OH(*υ*) sublayer is thought to gradually increase with vibrational level (Noll et al., 2016; von Savigny and Lednytskyy, 2013; von Savigny et al., 2012; Liu and Shepherd, 2006; McDade, 1991; López-Moreno et al., 1987), the exact opposite trend for *T*_cold_, i.e., a temperature decrease with increasing altitude, would be expected if the OH(*υ*) rotational temperatures were indeed in equilibrium with the local kinetic temperature.

In the literature, three often used OH Meinel bands for determining rotational temperatures are the (8–3), (6–2), and (3–1) bands. When temperatures of the aforementioned or other OH Meinel bands are reported in the literature, the observed differences are usually attributed to uncertainties associated with the employed techniques or the natural variability of mesospheric OH emissions; considering the possible role of non-LTE effects is a relatively rare occurrence (von Savigny et al., 2012; Dyrland et al., 2010).

**Figure 1:**
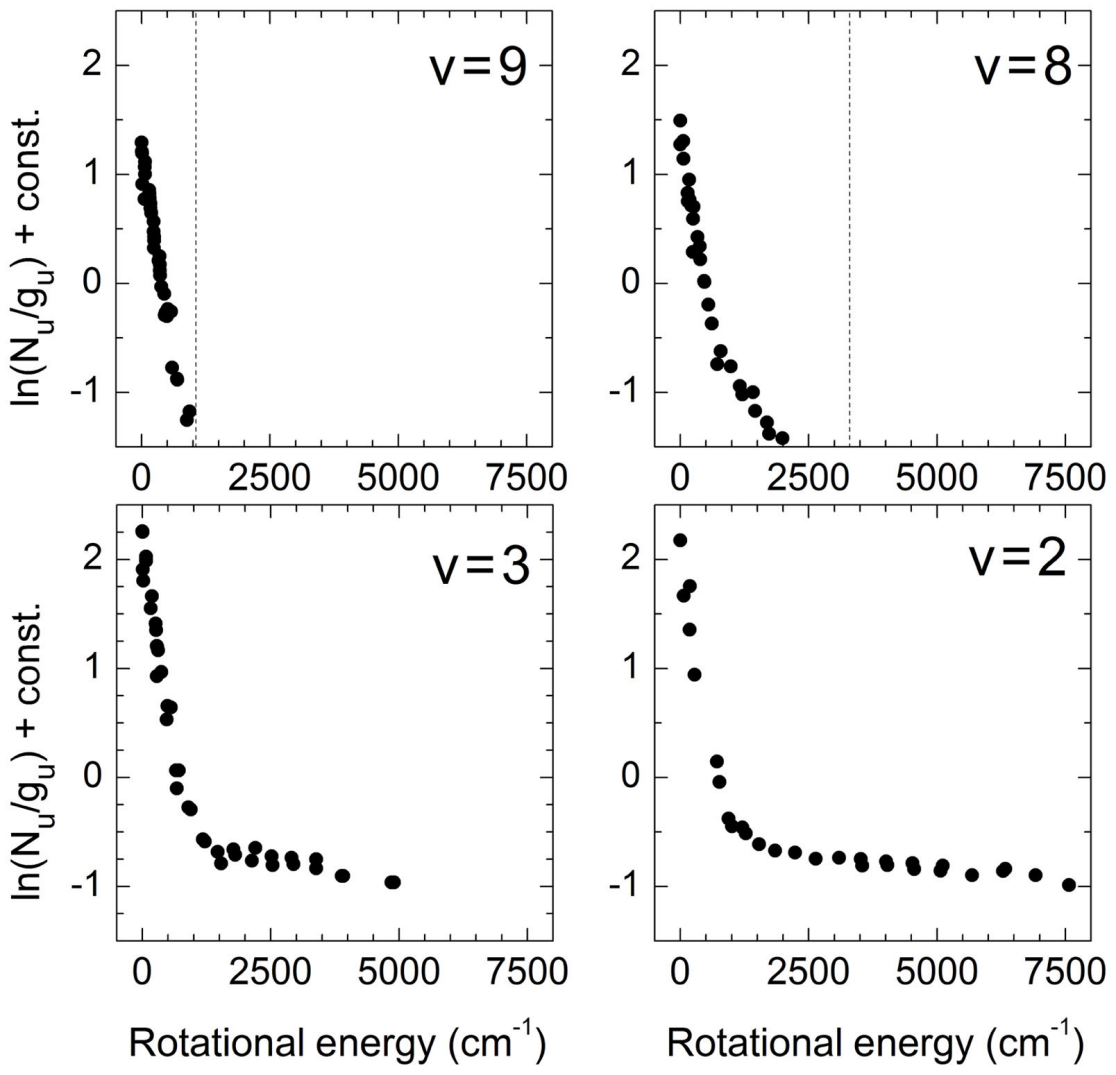
Mesospheric OH(*υ* = 2, 3, 8, and 9) rotational population distributions adapted from Fig. 2 of Oliva et al. (2015). Vibrational levels *υ* = 2 and 3 are the most near-resonant multi-quantum relaxation pathways for *υ* = 8 and 9, respectively, according to Eq. (2). The dashed lines show the nominal energetic limit for reaction H + O_3_.

Another set of relevant near-infrared observations highlighting the persistent high rotational excitation of mesospheric OH(*υ*) was reported by Oliva et al. (2015), who used the GIANO high-resolution spectrograph at the La Palma Observatory to obtain sky spectra in the wavelength range 0.97–2.4 μm. This group averaged data for 2 h at a resolution of ~ 36 000 in an attempt to provide a better characterization of the nightglow continuum and “sky suppression” for astronomical investigations. The results demonstrate high rotational excitation even for the lowest OH vibrational levels, in excellent agreement with the observations of Cosby and Slanger (2007). Figure 1 shows the rotational population distributions for levels *υ* = 2, 3, 8, 9 from the data set of Oliva et al. (2015). The H + O_3_ nominal energetic limit is indicated by dashed lines for *υ* = 8, 9. These two rotational population distributions are markedly different than those for *υ* = 2, 3, with the latter two displaying persistent tails of highly rotationally excited levels. We note that yet higher rotational levels in OH(*υ* = 2, 3) have been reported by Dodd and coworkers with rotational energies of 10 000 cm^−1^ and 12 000 cm^−1^, respectively (Dodd et al., 1993, 1994; Smith et al., 1992). The significantly larger radiative rates for the pure rotation–rotation transitions and the limb viewing geometry enabled Dodd and coworkers to sensitively detect signals for rotational transitions originating from levels as high as *N* = 33 (for vibrational levels *υ* = 0–3). As mentioned above, such high rotational excitation cannot be accounted for with dipole-allowed transition selection rules and requires an alternate source.

Noll et al. (2016, 2015) and Cosby and Slanger (2007) determined OH(*υ*) rotational temperatures by considering lines from a few low rotational quantum numbers. They determined *T*_cold_ by performing a fit to their truncated data set using a Boltzmann distribution for a single temperature. In contrast, Oliva et al. (2015) performed a two-temperature fit to all observed rotational lines regardless of quantum number and fixed the value of *T*_cold_ at a nominal mesospheric temperature of 200 K. A summary of the results from the previous studies of Cosby and Slanger (2007), Noll et al. (2015), and Oliva et al. (2015) is presented in Table 1. This table also includes additional information relevant to rotational relaxation near 90 km that will be discussed below.

A remarkable trend is evident from the rotational temperatures corresponding to high rotational levels, *T*_hot_, shown in the fifth column of Table 1, as determined by Oliva et al. (2015). *T*_hot_ rises dramatically as the vibrational level decreases, with values from approximately 1000 K for *υ* = 9 to ~12 000 K for *υ* = 2. We also highlight the different behavior of *T*_hot_ for vibrational levels *υ* = 7–9, in which more than 90 % of nascent OH(*υ*) is produced following the H + O_3_ reaction when compared to the behavior of the lowest vibrational levels that exhibit the most extreme *T*_hot_ values. The sixth column of Table 1 shows the radiative lifetime of OH(*υ*), *τ*_rad_, based on the most recent study by Brooke et al. (2016). The seventh column presents the calculated number of collisions during *τ*_rad_, and the last one shows estimates for the product of *τ*_rad_ multiplied by an estimate of the pressure near 90 km (e.g., NRLMSISE-00 model; Picone et al., 2002).

In their experimental study, Kliner and Farrow (1999) found that OH translational relaxation in N_2_ and O_2_ occurred very rapidly within a value of (*P* × *τ*_rad_) of ~ 27 Pa μs. In stark contrast, they found that complete rotational equilibration of OH(*υ* = 0, *N* ≤ 12) requires a value of (*P* × *τ*_rad_) that is approximately 70 times larger, ~ 1.9 kPa μs. Even after correction for the temperature difference between the experiments of Kliner and Farrow and the mesosphere, the (*P* × *τ*_rad_) values shown in Table 1 are significantly smaller than 1.9 kPa μs for all listed OH(*υ*) vibrational levels. We note here that the higher the rotational excitation, the slower the rate of rotational relaxation, and much higher rotational levels than *N* = 12, up to *N* = 33, have been observed for mesospheric OH(*υ* = 0–3) (Smith et al., 1992). Therefore, the calculated values of *P* × *τ*_rad_ in Table 1 provide another clear indication of non-local equilibrium conditions for OH(*υ*).

From Table 1, we also note that the number of collisions experienced by OH(low-*υ*) before emission is significantly larger than that experienced by OH(high-*υ*). This is consistent with the expectation that the lowest OH vibrational levels are closer to thermal equilibrium, especially for the lower parts of the mesosphere. For low-*N* and low-*υ* levels, the radiative relaxation rate by pure rotational transitions is also significantly smaller than that for rovibrational transitions, and both rates are smaller than the rate of collisional relaxation (Dodd et al., 1994). However, because of the existence of multi-quantum relaxation pathways that connect high and low vibrational levels, a fraction of the rotational population distribution is always in non-LTE, as demonstrated in Fig. 1.

Another important piece of evidence for the characterization of the OH rotational temperatures as non-LTE for all observed levels stems from the fact that in the studies by Noll et al. (2016) and Cosby and Slanger (2007), consideration of additional rotational levels beyond the lowest three resulted in gradually different results for the OH rotational temperature. For example, Noll et al. (2016, 2015) reported that the determined OH rotational temperature increased by 1 K on average when the analysis considered the *P*_1_ (*N* = 4) line together with the first three *P*_1_-branch lines. For the analysis of the *P*_2_-branch lines, use of the first four rotational lines of this branch resulted in an average increase in the rotational temperatures by 11 K when compared to the reference set of the first three *P*_1_-branch lines (Noll et al., 2016).

To further support the conclusions of this report, we reanalyzed the data set of Oliva et al. in two different ways. First, we followed a similar approach to that of Cosby and Slanger (2007) and truncated the Oliva et al. data set to only include low OH rotational lines originating from levels with rotational energy less than 500 cm^−1^. We then performed fits to a simple Boltzmann distribution, which will heretofore be referred to as single-temperature fits. Second, we slightly varied the approach of Oliva et al. by performing two-temperature fits with both *T*_cold_ and *T*_hot_ as unconstrained adjustable parameters. For the results reported here we used the most recent set of transition probabilities available in the literature (Brooke et al., 2016; abbreviated as BBW16). Analysis of a data subset using the transition probabilities of van der Loo and Groenenboom (2008, 2007) yielded results that are very similar for the bands reported by Oliva et al. The results of our analysis using BBW16 are summarized in Table 2.

**Figure 2:**
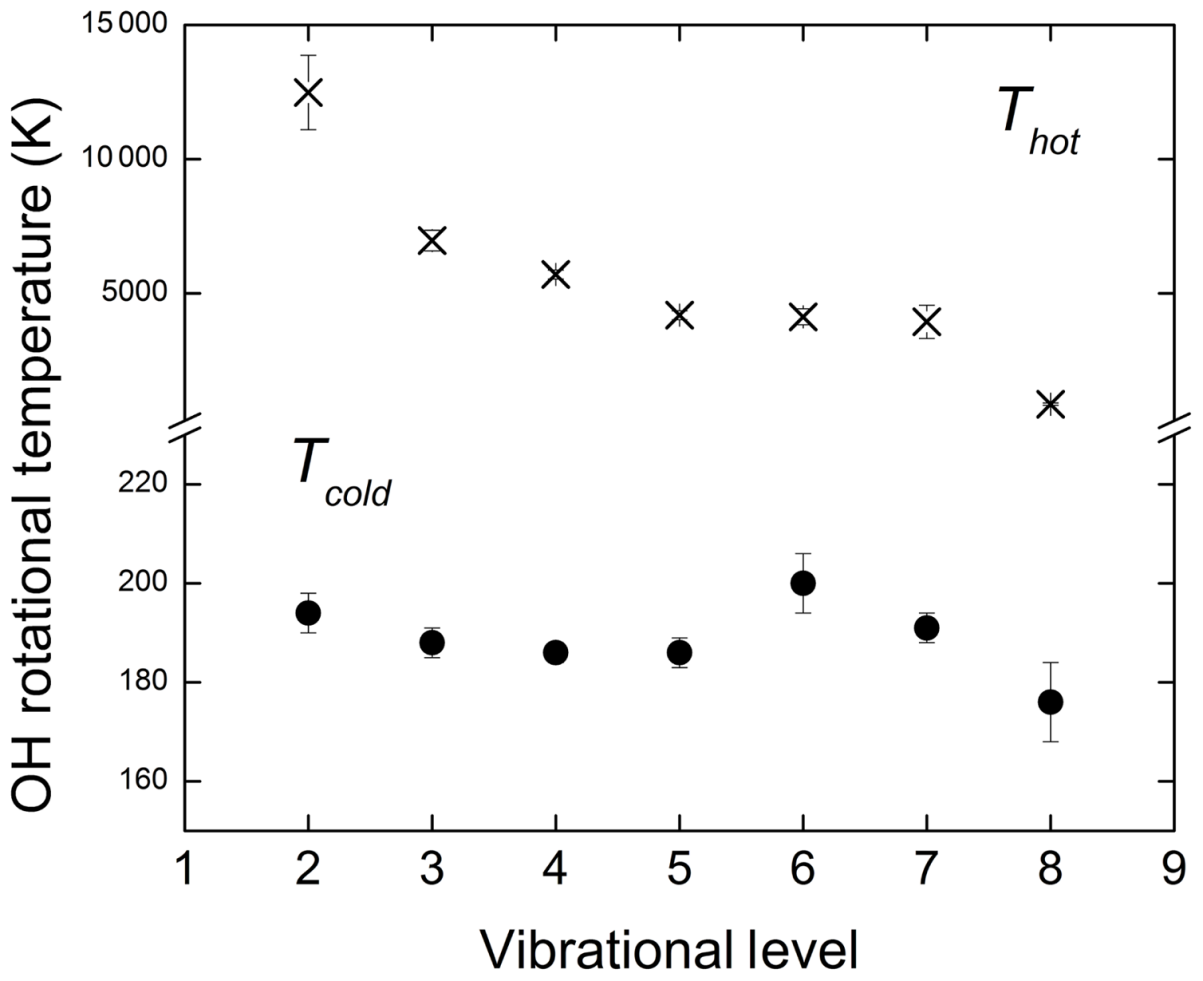
Results for OH(*υ*) *T*_cold_ (solid circles) and *T*_hot_ (slanted crosses) rotational temperatures obtained from simultaneous two-temperature fits of the data set reported by Oliva et al. (2015).

Figure 2 shows the results for *T*_cold_ and *T*_hot_ from our analysis of the Oliva et al. (2015) data set. The most striking finding is that the two-temperature fit generates significantly different results for the values of *T*_cold_. Postulating a Boltzmann distribution at an elevated temperature *T*_hot_ has an effect on the population distribution with low rotational excitation that determines the value of *T*_cold_. In the case of the single-temperature fits, the contributions from the non-LTE rotational population distribution are not subtracted from the observed population at low rotational quantum levels. As shown in Table 2 for the data set by Oliva et al., this results in values of *T*_cold_ from two- and single-temperature fits that differ by as much as Δ*T*_cold_ = −28 K for OH(*υ* = 8). Therefore, to determine accurate rotational temperatures it is essential to take into account the non-LTE contributions for each OH(*υ*) vibrational level. Clearly, the fraction of the population that is in highly rotationally excited levels and the “temperature” of this non-LTE distribution will influence the extent to which the determination of *T*_cold_ will be affected. The present results suggest that OH temperature measurements observing one of the lowest vibrational levels might be least affected by the non-LTE effects on *T*_cold_ under certain atmospheric conditions, for example the altitude and distribution of the atomic oxygen layer. Regardless, this cannot be assumed to be the case without a detailed understanding of the processes that give rise to OH rotational excitation as the nascent rovibrational population distribution relaxes and of the most adequate form to describe the effects of that relaxation.

It follows that a reassessment of OH rotational temperature measurements reported to date is warranted. Detailed analysis of existing or future high-resolution data sets that encompass a wide range of rotational levels is needed, including systematic checks of the extent to which a two-temperature Boltzmann distribution is an adequate representation of the rotational population distribution for all OH(*υ*) vibrational levels, a detailed consideration of the selection of individual rotational lines and their effect on estimated uncertainties, and a critical evaluation of all available sets of OH transition probabilities.

In summary, the most recent high-resolution studies of OH(*υ*) nightglow involving simultaneous OH(*υ*) observations and using different astronomical telescopes consistently demonstrate that the rotational temperatures determined from the lowest rotational levels, *T*_cold_, are affected by non-LTE effects. The rotational population distributions have extremely hot tails and the rotational temperatures determined for high rotational levels, *T*_hot_, increase rapidly as the vibrational quantum number decreases. The available laboratory evidence from studies on OH rotational energy transfer also indicates that, under mesospheric conditions, thermalization of the OH rotational population distributions for all vibrational levels is not complete. Therefore, the rotational temperatures routinely determined from mesospheric OH(*υ*) observations cannot be generally assumed to reflect the local kinetic temperature.

## 6 OH rotational temperatures and multi-quantum relaxation

We will now consider the role of OH(*υ*) multi-quantum vibrational relaxation as a possible driver of non-LTE conditions. The multi-quantum vibrational relaxation process of Eq. (2) provides an efficient means that directly connects high and low OH vibrational levels in the presence of O atoms. This finding addresses a long-standing discrepancy between OH(*υ*) atmospheric observations and model calculations, with the models underestimating the number density of low vibrational levels unless additional processes that produce OH(low-*υ*) are invoked. One such example, mentioned above, is the deficit that Dodd et al. (1994) encountered in their “chemical production” model. Other models have also encountered similar difficulties (Grygalashvyly et al., 2014; Grygalashvyly, 2015, and references therein).

Just as important, the multi-quantum vibrational relaxation of Eq. (2) may be a source of rotational excitation for OH(low-*υ*). The process of Eq. (3) involving the OH(*υ* = 9) */* OH(*υ* = 3) pair is exothermic by Δ*E* = −118 cm^−1^, assuming the O(^3^*P*_2_) ground state is involved. This is the most near-thermoneutral vibrational level combination for this process in the OH(*υ*) vibrational manifold, followed by the OH(*υ* = 5) */* OH(*υ* = 0) pair (Δ*E* = −343 cm^−1^). The OH(*υ* = 8) */* OH(*υ* = 2) combination is exothermic by Δ*E* = −1122 cm^−1^, the OH(*υ* = 7) */* OH(*υ* = 1) by Δ*E* = −2111 cm^−1^, and the OH(*υ* = 6) */* OH(*υ* = 0) pair by Δ*E* = −3095 cm^−1^. If the final vibrational quantum number is smaller than the most near-resonant paths shown above, the resulting exothermicity values are far greater. The relative propensity of these processes has not yet been investigated. In all cases, however, the energy released in the relaxation process is partitioned between translational energy and rotational excitation. Thus, as the multi-quantum relaxation OH(high-*υ*) + O generates lower vibrational levels, the result will be additional kinetic and rotational excitation, an observation consistent with the behavior seen for *T*_hot_ in Table 1. Because of this process and other possible multi-quantum relaxation pathways involving O_2_ and N_2_ (McCaffery et al., 2011; Adler-Golden, 1997; Dodd et al., 1994) that could lead to the production of rotationally excited OH(low-*υ*), it is insufficient to think of the observed OH(low-*υ*) rotational levels only as nascent rovibrationally excited OH that has gradually relaxed and equilibrated through radiation and several collisions.

## 7 Discussion

We begin with some remarks relevant to the interpretation of mesospheric OH rotational temperatures. Because not only high rotational levels, but also the lower ones, are not in complete LTE it is essential to conduct simultaneous measurements that resolve transitions from low and high vibrational and rotational levels to develop a deeper understanding of the observed non-LTE behavior. The fine details of the variability and vibrational level dependence exhibited by *T*_cold_ and *T*_hot_ for mesospheric OH can be attributed to the complicated reaction and collisional relaxation dynamics of the relevant OH(*υ*) production and removal mechanisms. These are not yet fully understood, notwithstanding the fact that the recently established fast OH(*υ*) + O multi-quantum V–E pathway represents an important new insight. Additional studies of this type are required to probe the short- and long-term variability and better understand the dynamics of non-LTE conditions in the mesosphere.

The next point to highlight is that the steep gradient in the number density of mesospheric atomic oxygen guarantees strong altitude dependence for these multi-quantum vibrational relaxation pathways. For example, the number density of O atoms is approximately 1 % of that of the O_2_ near 88 km and increases by about a factor of 3 to approximately 10 % of the O_2_ number density near 95 km (e.g., NRLMSISE-00 model; Picone et al., 2002). It is also well established that atomic oxygen exhibits rich variability that, combined with the very different OH(*υ*) removal rate constants by O and O_2_, is expected to lead to highly complex behavior.

Cosby and Slanger (2007) reported that the temperature difference Δ*T*_9_*_,_*_3_ = *T* (*υ* = 9) – *T* (*υ* = 3) in one of the studied data sets increases as the [OH(*υ* = 3)] */* [OH(*υ* = 9)] intensity ratio decreases during the night; i.e., Δ*T*_9_*_,_*_3_ increases as the relative efficiency of collisional relaxation from *υ* = 9 to *υ* = 3 decreases. It is tempting to attempt an explanation for this behavior invoking a possible role for the new multi-quantum OH(*υ*) + O relaxation pathway and variations in the O atom number density in the mesosphere during the night (e.g., Smith et al., 2010). As the airglow layer conditions change during the night, a number of multi-quantum relaxation processes vary in importance and contribute accordingly to the observed Δ*T*_9_*_,_*_3_ values. These phenomena deserve further exploration and underscore the importance of understanding the observations in terms of the detailed atomic and molecular processes involved.

From the discussion above it is evident that the collisional relaxation dynamics of mesospheric OH are complex and variable. The recent developments regarding the OH(high-*υ*) + O fast, multi-quantum vibrational relaxation that is coupled to the CO_2_ 4.3 μm emission represent significant advances (Panka et al., 2017b; Kalogerakis et al., 2016; Sharma et al., 2015). At the same time, it is also clear that our understanding of the relevant details requires further refinement. Considering the large amount of extant data from mesospheric ground- and space-based observations, it is essential that synergistic theoretical, modeling, and laboratory studies continue to address the remaining gaps in our knowledge. Recent measurements demonstrate that the OH(*υ*) rotational temperatures determined from observations are partially equilibrated effective rotational temperatures, even for the lowest rotational levels studied. Most important, the emerging vision for the future is that by developing a detailed understanding at the atomic and molecular level of the mechanisms that control mesospheric non-LTE processes it will be possible to determine O atom densities and altitude profiles from simultaneous, high-resolution, ground-based observations. The new multi-quantum OH(*υ*) + O vibrational relaxation processes open exciting new research opportunities to probe and understand the variability of mesospheric OH(*υ*) non-LTE conditions.

## 8 Conclusions

The available evidence from laboratory experiments and recent observations involving simultaneous OH(*υ*) measurements at high resolution demonstrates that mesospheric OH(*υ*) rotational temperatures cannot be generally assumed to correspond to the local kinetic temperature regardless of the vibrational or rotational level. The observed steady-state rotational population distributions of mesospheric OH(*υ*) exhibit pronounced hot tails that are not fully thermalized. The recently established fast multi-quantum vibrational relaxation of OH(high-*υ*) by O atoms efficiently populates OH(low-*υ*) levels and enhances rotational excitation. The quantitative details of the processes involved require additional synergistic investigations by observations, modeling and theoretical calculations, and laboratory experiments. The multi-quantum vibrational relaxation of OH(*υ*) opens a new window into the details of mesospheric non-LTE conditions and the possibility to develop novel diagnostic tools for monitoring O atoms in this region of the atmosphere.

## Supplementary Material

Oliva Data Used

## Figures and Tables

**Table 1:** OH(*υ*) rotational temperatures reported in studies of high-resolution astronomical sky spectra and information relevant to collisional relaxation near 90 km.

Vibrational Level (*υ*)	*T*_cold_ CS07^a^ (K)	*T*_cold_ NKK15^b^ (K)	*T*_cold_ OOS15^c^ (K)	*T*_hot_ OOS15^c^ (K)	Radiative Lifetime^d^ (ms)	Collisions in *τ*_rad_^e^	Product *P* × *τ*_rad_^f^(Pa μs)
9	203.1 ± 1.9	196.5 ± 1.1	200 (fixed)	1000	5.77	58	120
8	212.3 ± 2.6	202.0 ± 1.0	200	1300	6.75	67	133
7	205.6 ± 3.2	194.2 ± 1.1	200	1600	8.12	81	160
6	209.9 ± 4.8	196.7 ± 1.1	200	4000	10.01	100	200
5	205.3 ± 3.6	194.0 ± 1.1	200	4200	12.54	125	253
4	203.1 ± 3.5	195.0 ± 1.1	200	7000	16.07	161	320
3	196.0 ± 5.4	191.9 ± 1.3	200	7000	21.40	214	427
2	–	187.6 ± 1.9	200	12 000	31.20	312	627

a Cosby and Slanger (2007); single observation 3MAR00 05:39 UT; transition probabilities from Goldman et al. (1998); single-temperature fit using low rotational lines.

b Noll et al. (2015); averaged results from 343 spectra, each containing 25 OH bands; transition probabilities from the HITRAN2012 database; Rothman et al. (2013); single-temperature fit using low rotational lines.

c Oliva et al. (2015); averaged data for 2 h; transition probabilities from van der Loo and Groenenboom (2008); two-temperature fit of all rotational lines with *T*_cold_ fixed at 200 K.

d Brooke et al. (2016).

e Based on a collision frequency of 10^4^ Hz, estimated from typical total number densities encountered at 90 km (NRLMSISE-00 model; Picone et al., 2002).

f Based on a pressure estimate for 90 km with a value of 0.2 Pa (NRLMSISE-00 model; Picone et al., 2002).

**Table 2:** OH(*υ*) rotational temperatures *T*_cold_ and *T*_hot_ for low and high rotational quantum numbers, respectively, from our reanalysis of the data reported by Oliva et al. (2015). Fit results for a single temperature (*T*_cold_) and for two temperatures (*T*_cold_ and *T*_hot_) are shown using the most recent set of transition probabilities by Brooke et al. (2016) together with the difference between the values determined by the two different fit types.

Band (*υ*′, *υ*″)	Single-temperature fits (Stfs)	Two-temperature fits (Ttfs)		Δ*T*_cold_

	*T*_cold_ (K)	*T*_cold_ (K)	*T*_hot_ (K)	*T*_Ttf_–*T*_Stf_ (K)
(9,7)	189 ± 4	–	–	–

(8,6)	204 ± 6	176 ± 8	869 ± 48	−28
(7,4)	203 ± 3	191 ± 3	3936 ± 620	−12
(6,4)	195 ± 2	200 ± 6	4124 ± 302	+5
(5,3)	192 ± 3	186 ± 3	4182 ± 165	−6
(4,2)	188 ± 2	186 ± 2	5700 ± 167	−2
(3,1)	188 ± 3	188 ± 3	6966 ± 387	0
(2,0)	183 ± 10	194 ± 4	12 483 ± 1389	+11
